# In-Process Analysis of Melt Pool Fluctuations with Scanning Optical Coherence Tomography for Laser Welding of Copper for Quality Monitoring

**DOI:** 10.3390/mi13111937

**Published:** 2022-11-09

**Authors:** Thomas Will, Tobias Jeron, Claudio Hoelbling, Lars Müller, Michael Schmidt

**Affiliations:** 1 Institute of Photonic Technologies, Friedrich-Alexander Universität Erlangen-Nürnberg, 91052 Erlangen, Germany; 2 Erlangen Graduate School in Advanced Optical Technologies (SAOT), Friedrich-Alexander Universität Erlangen-Nürnberg, 91052 Erlangen, Germany; 3 Vitesco Technologies Germany GmbH, 90411 Nürnberg, Germany

**Keywords:** optical coherence tomography, interferometry, laser welding, copper, process monitoring, feature engineering, machine learning, quality monitoring, melt pool

## Abstract

Optical coherence tomography (OCT) is an inline process monitoring technology for laser welding with various applications in the pre-, in-, and post-process. In-process monitoring with OCT focuses on the measurement of weld depth by the placement of a singular measurement beam into the keyhole. A laterally scanned measurement beam gives the opportunity to measure the keyhole and melt pool width. The processing region can be identified by separating higher signal intensities on the workpiece surface from lower signal intensities from the keyhole and the melt pool. In this work, we apply a scanned measurement beam for the identification of keyhole fluctuations. Different laser processing parameters are varied for laser welding of copper to evoke welds in the heat conduction regime, stable deep penetration welding, and unstable deep penetration welding. As keyhole instabilities can be related to the generation of spatter and other defects, we identified a feature for the classification of different weld statuses. In consequence, feedback can be given about possible defects which are originated in keyhole fluctuations (e.g., spatter).

## 1. Introduction

Optical coherence tomography (OCT) is an interferometric distance measurement technology and can be used as a versatile inline process observation tool for laser welding processes. Three laser process observation stages can be differentiated for quality monitoring [1], pre-, in-, and post-process observation. The pre-process observation enables the measurement of the joint position for accurate positioning of the processing laser [2,3,4]. Post-process observation focuses on the identification of undesired weld quality by observation of the solidified weld geometry [5,6]. The in-process observation stage addresses aspects such as welding stability [7] and weld depth measurement [8,9,10].

The possibility to measure the keyhole depth and hence estimate the weld penetration depth is a key advantage of OCT in comparison to alternative measurement technologies [11]. The interferometric measurement of the keyhole depth is possible by the placement of a measurement beam in the keyhole bottom [8]. A perpendicular scanning of the measurement beam relative to the welding direction would increase the geometrical measurement information as information about the melt pool width could be extracted. 

Welding instabilities can be identified by observation of the melt pool and can give feedback on different welding quality characteristics [1]. Welding instabilities are based on the instability of the keyhole and lead to undesired weld characteristics such as melt ejections and pore formation [12,13]. These keyhole fluctuations are closely related to the morphology of the molten pool [14]. Process observations of the interaction region with its molten pool are possible in 2D with different camera setups and in 1D with photodiodes.

A photodiode records the intensity of electromagnetic radiation from the process zone over time. Information about the weld stability can be given from photodiodes that are sensitive in the wavelength region of the processing laser or that are sensitive in the infrared (IR) wavelength region. Instabilities in the process zone can be identified with the identification of increasing fluctuations in the reflected signal intensity [15]. This is the case, as stable keyholes result in fewer fluctuations of reflected light than an unstable keyhole [16]. The analysis of IR radiation can give feedback on weld instabilities by analysis of the thermal radiation from the interaction region [16] but does not allow to separate measurement information from the melt pool or the vapor plume. 

IR cameras measure the temperature profile of the interaction region [17] and, hence, can differ between vapor plume, keyhole, and melt pool. In consequence, IR camera measurements can indirectly conclude the weld pool width and length to observe the welding stability [18]. Cameras operating in the visible (VIS) wavelength range use an additional light source for weld pool illumination and can extract the melt pool geometry by intensity variations of the grayscale images [19]. 

OCT is a potential technology that might enable the simultaneous measurement of the weld penetration depth and melt pool geometry by perpendicular scanning of the measurement beam. This should be possible as two attributes of OCT can be used. First, the interferometric distance measurement coaxial to the processing laser. Second, the resulting signal intensity changes for measurements on the workpiece surface and the interaction region [2]. However, current literature neither describes OCT’s capability of measuring in-process weld instabilities nor does it elaborate on its limitations and potential.

Our work focuses on two key aspects. First, the description of our methodological approach to extract relevant features from a laterally scanned measurement beam in the keyhole region. Second, the discussion of measurement results with regards to the discriminability of welding status (e.g., stable deep penetration welding, unstable deep penetration welding, heat conduction welding), the impact of laser processing parameters, and the limitations of this measurement approach with regard to OCT settings.

## 2. Materials and Methods

First, the experimental setup is introduced. Second, the experimental procedure is described. Lastly, the data processing approaches are presented for the OCT weld width measurement from laterally scanned OCT measurements and methods for the analysis of the measurement results.

### 2.1. Experimental Setup

**Figure 1:**
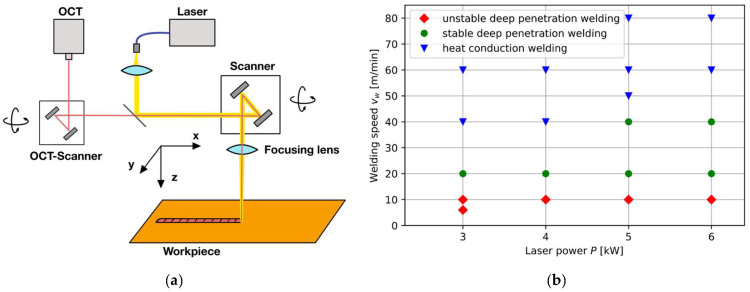
(**a**) Schematic experimental setup with OCT, processing laser, programmable focusing optics, and workpiece; (**b**) Overview of laser welding parameters depending on weld category (unstable deep penetration welding (red), stable deep penetration welding (green), heat conduction welding (blue).

The experimental setup consists of programmable focusing optics with cross-jet, fiber-coupled processing laser, and OCT (Figure 1a). The laser welding process is carried out with a continuous wave commercial disk laser (Trumpf TruDisk 6001, Trumpf, Ditzingen, Germany). The processing laser has a wavelength of 1030 nm with a maximum average power of 6000 W. The laser beam is delivered by an optical fiber with a core diameter of 100 µm into programmable focusing optics (Trumpf PFO 33-2). The focusing optics have a focal length of 255 mm and result in a laser spot diameter of 170 µm. The programmable focusing optic consists of galvanometer scanners and allows for scanning in an elliptical field of 90 mm × 50 mm. The OCT is attached to the programmable focusing optics and enables a coaxial positioning of the measurement beam. The OCT is an SD-OCT with a superluminescent diode with a central wavelength at 840 nm and a bandwidth of 40 nm. The measurement beam is detected on a 2048-pixel line sensor with a maximum measurement frequency of 70 kHz. The OCT system has an axial resolution of 12 µm in the z-direction and a lateral resolution of 25 µm in the y-direction.

### 2.2. Experimental Procedure

A weld seam with a length of 60 mm is welded bead-on-plate on pure copper workpieces (Cu-OFE, 70 mm × 30 mm × 5 mm) in the focus position (z = 0). The OCT measurement beam is scanned perpendicular to the weld seam in the x-direction according to the welding speed. The OCT measurement line has a length of 2 mm in the y-direction with 200 measurement points and resulted in a frame rate of 350 Hz (Figure 1a). The pixel size in the y-direction is 10 µm according to the number of measurement points and measurement beam length.

Processing parameters are varied for laser process parameters (Figure 1b), and OCT calibration parameters are given in Table 1. The laser process parameters are varied by process regime in the case of heat conduction welding, stable deep penetration welding, and unstable deep penetration welding with the formation of melt ejections. The combination of laser power and welding speed is adjusted to the process regime under investigation and includes parameter values between 3000 W and 6000 W as well as 6 m/min and 80 m/min (also Figure 1b). Here, unstable deep penetration welding is identified by the existence of melt ejections as well as resulting height depositions and underfill. Humping is determined with visual inspection and metallographic analysis. Heat conduction welding is separated from stable deep penetration welding by the calculation of the aspect ratio between metallographic weld depth and metallographic weld width. The OCT parameters are varied in terms of the measurement position in the x-direction relative to the tool center point (TCP), frame rate, and exposure time. The relative OCT position is chosen to be trailing due to the expected tilt of the keyhole wall and is located in the keyhole region. The measurement position is varied from 0.06 mm up to 0.12 mm in 0.02 mm steps, as keyhole mapping in literature resulted in optimal positioning for this step size [9]. The frame rate of the OCT image acquisition is varied between 87 Hz and 350 Hz for the identification of measurement-related limitations in the discriminability of different weld statuses. The exposure time of OCT’s line sensor is altered between 3 µs and 10 µs to analyze the impact of different exposure times on the measurement signal. Changes in the measurement signal are expected as measurement information from the interaction region is enhanced. Each parameter set defined in Table 1 is repeated three times.

### 2.3. Data Processing

**Figure 2:**
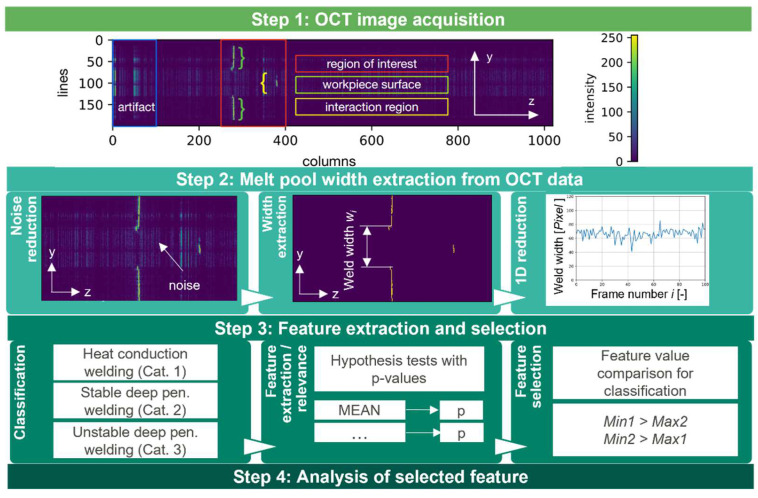
Four data processing steps for the identification of a relevant feature for the classification of laser process regimes based on OCT weld with measurements.

The OCT data processing is conducted in three main steps: OCT image acquisition, OCT weld width extraction, feature extraction, and selection. The fourth step is the analysis of the selected feature for weld quality analysis which is further elaborated in Section 3 (Figure 2).

The acquired OCT image consists of relevant information in the region of interest (ROI) and irrelevant measurement information (e.g., artifacts). The ROI contains the measurement information of the workpiece surface and the interaction region, whereas the outer area of the ROI may contain artifacts from the DC-term [2]. The second step of the data processing focuses on the weld width extraction from the OCT data and begins with noise reduction. Here, the measurement information within the first rows is cropped to avoid any influence from artifacts. The width extraction is performed by counting the number of measurement points along the measurement line that do not correspond to the workpiece surface. Thresholding is applied to support the separation of measurement information from the interaction region from the workpiece surface. The width information of each OCT image can be considered as a width time series along the weld direction. The presence of time-series data allows for supporting the feature extraction and selection with the FRESH algorithm [20] to identify significant features of time-series data for the classification task. The classification task aims to identify desired or undesired weld status based on welding stability. In consequence, the welding results are categorized for FRESH according to heat conduction welding (hcw), stable deep penetration welding (sdpw), and unstable deep penetration welding (udpw). In consequence, 276 relevant features are detected by FRESH with the help of hypothesis tests [21]. However, not all significant features enable a perfect discriminability of welding results. In consequence, a further reduction of the number of features is performed by filtering with the following condition:

(1) Min1>Max2 ∧Min2>Max1.

Features are only relevant when the comparison of feature values between two categories results in feature values where the minimum of category 1 *Min*1 is bigger than the maximum of category 2 *Max*2 as well as the minimum of category 2 *Min*2 is bigger than the maximum of category 1 *Max*1. The unambiguous discriminability of three categories is only possible with two features. The first feature is the sum value of all widths, whereas the second feature is the absolute energy *ae_w_*:

(2) $$ae_{w}=\sum_{i=0}^{n_{scan}}w_{i}^{2}.$$

The absolute energy value of the time series *ae_w_* can be calculated from the sum value of all squared width values *w_i_* and hence is highly correlated with the sum value of all widths. The longer the time series *n_scan_* and the higher the measured OCT weld width *w_i_*, the higher the *ae_w_* value. The influence of high measured widths is increased by squaring its value, while the influence of low widths is reduced. A stronger impact of the measured width is desired to observe variations in the measured width based on process parameters. In consequence, the results are discussed with the feature absolute value *ae_w_*. Metallographic cross-sections are considered to analyze the impact on the resulting weld cross-section.

## 3. Results and Discussion

The goal of our work focuses on showing the discriminability of welding status based on OCT width measurements of the interaction region. Afterward, we discuss the impact of laser processing parameters on the identified feature values and the possible limitations of this measurement approach.

### 3.1. Classification of Welding Status

**Figure 3:**
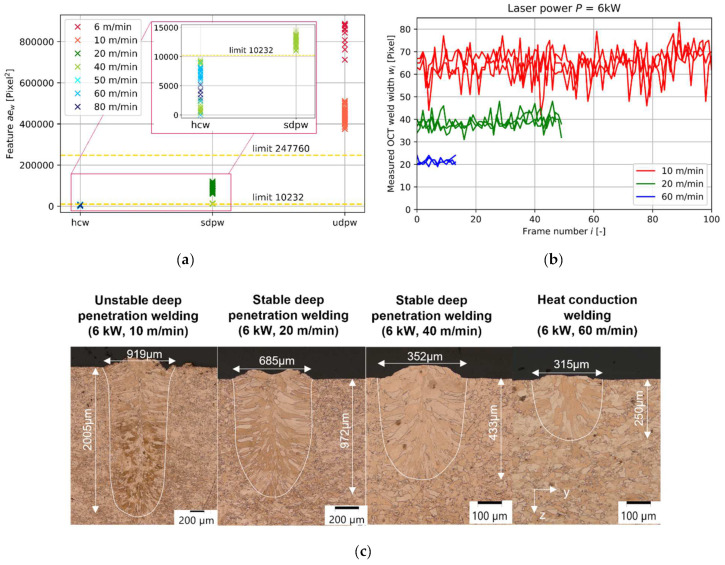
(**a**) Feature value for the three possible welding statuses: heat conduction welding (hcw), stable deep penetration welding (sdpw), and unstable deep penetration welding (udpw). The yellow dotted line indicates identified limits of each welding status; (**b**) Measured OCT weld width in the interaction region over frame number of OCT images for three different welding speeds at a constant laser power of 6 kW. Each speed corresponds to a process regime (hcw, 60 m/min) (sdpw, 20 m/min) (udpw, 10 m/min); (**c**) Metallographic cross-sections for four different welding speeds at a constant power of 6 kW.

Figure 3a shows the resulting feature value *ae_w_* for the three possible welding statuses: heat conduction welding, stable deep penetration welding, and unstable deep penetration welding. The yellow dotted line indicates identified feature limits of each welding status. A discriminability is given for all three categories and hence shows a proof-of-concept for the welding status identification based on the measured width of the interaction region. The lowest feature values can be found for hcw, increase for sdpw, and result in the highest feature values for udpw. This feature value trend originated in the measured OCT weld width and number of available OCT images (Figure 3b).

Heat conduction welding results in the lowest feature values as hcw shows the lowest number of frames in comparison to deep penetration welding due to the higher welding speed (Figure 3b). Additionally, the measured OCT weld width *w_i_* is generally smaller for hcw in comparison to deep penetration welding. This measurement result correlates well with metallurgical weld width measurements (Figure 3c). In consequence, hcw results can be expected in the feature range from 0 up to 10,232. Latter feature value results from the mean value of the lowest measured feature value in sdpw and the highest feature value in hcw.

In stable deep penetration welding, the feature values are higher in comparison to feature values for hcw as higher measured OCT weld widths can be found, and lower welding speeds are employed (Figure 3b). Unstable deep welding results in the highest measured feature values. This is the case as the highest measured OCT weld width can be found for udpw in comparison to the other process regimes, as well as the lowest employed welding speeds (compare Figure 3a and Figure 3b). The time series for udpw show stronger fluctuations in the measured OCT weld width in comparison to sdpw and hcw. Stronger fluctuations in the measured signal are expected for udpw due to a highly dynamic keyhole and melt pool behavior [12]. The limit for classification of sdpw and udpw is set to 247,760 and results from the mean value of the lowest feature value in udpw and the highest feature value in sdpw. Feature values above 247,760 can be classified as udpw.

In conclusion, the resulting discriminability of welding status is possible with the feature value *ae_w_* due to the strong impact of the welding speed on the weld result, as the feature value strongly depends on the number of OCT-acquired images. The exact feature value limit for classification may vary depending on the OCT set processing and measurement parameters, as other OCT and laser welding parameter combinations may impact the resulting feature value. How these parameters may impact the resulting feature value is elaborated in the next chapters.

### 3.2. Influence of Laser Welding Speed on Weld Width Feature

**Figure 4:**
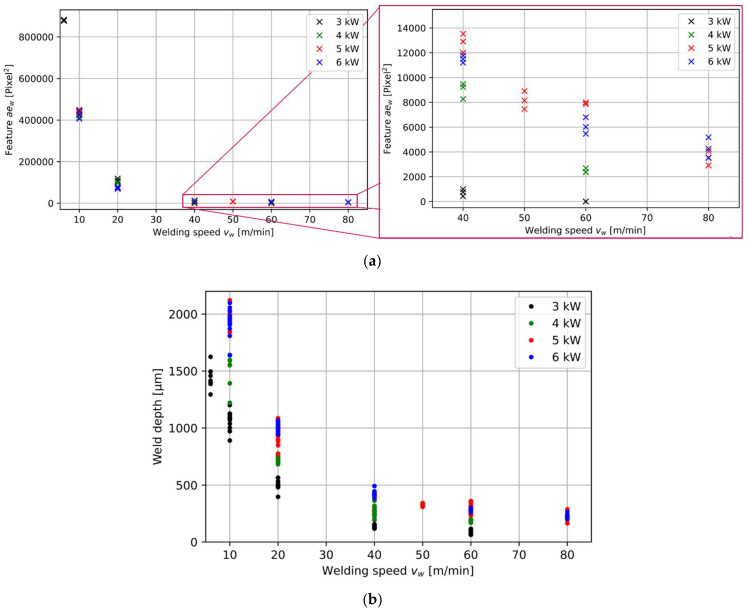
(**a**) Feature value over welding speed for different laser powers; (**b**) Metallurgical weld depth over welding speed for different laser powers.

The laser welding speed is the main influencing parameter on the feature value and shows a reciprocal correlation with the feature value *ae_w_* (Figure 4). The lower the laser welding speed, the higher the identified feature value. The higher the feature value, the higher the measured OCT weld width. This finding can be connected to the identified increase in metallurgical weld width and weld depth within metallographic cross-sections (Figure 3c). This is in accordance with findings in the literature [19], where an increase in measured melt pool width is directly correlated with an increase in the resulting weld width. 

**Figure 5:**
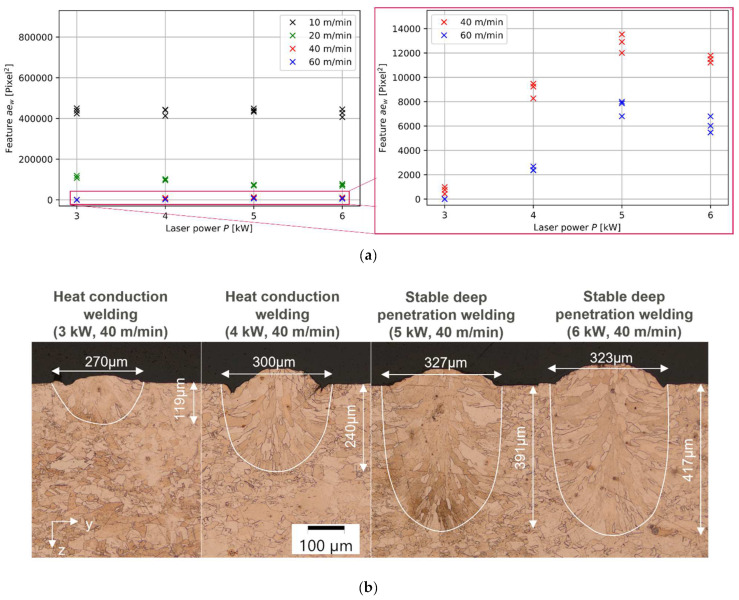
(**a**) Feature value over laser power for different laser welding speeds; (**b**) Metallographic cross-sections for different laser powers at a constant laser welding speed of 40 m/min.

The feature value can also be connected with the measured metallurgical weld depth (compare Figure 4a and Figure 4b). Here, the measured metallurgical weld depth follows the reciprocal behavior of the feature value over the welding speed. This is in accordance with earlier findings [22] that found a proportionality between weld depth and weld width. In consequence, an indirect estimation of the weld depth can be taken from the OCT width measurements and gives rise to further future work. However, even though the identified feature value follows a similar behavior to the welding speed, such as the measured metallurgical weld depth, we can not confirm the same behavior for different laser powers. Higher laser power can be connected with an increase in weld depth (Figure 5b). This behavior cannot be confirmed for feature values at 20 m/min (Figure 4a). In consequence, we expect that OCT weld width measurements do not allow to con-clude on the weld depth as accurate as keyhole depth measurements [11]. A more thorough analysis of the impact of the laser power on the resulting feature values can be found in the following.

### 3.3. Influence of Laser Power on Weld Width Feature

Changes in laser power do not result in significant changes in feature values in contrast to changes in laser welding speed (compare Figure 4 and Figure 5a). Especially, welds that are categorized as udpw do not show an influence of the laser power on the feature value (Figure 5a, 10 m/min). Presumably, the unstable weld condition does not allow for the identification of laser power-related variations. 

Stable deep penetration welding also shows stable feature values for different power levels (e.g., 5 kW and 6 kW with 40 m/min and 20 m/min) (Figure 5a). In the case of welding at a welding speed of 40 m/min, it can be seen that the feature value increases or shrinks according to the resulting metallurgical weld width for welds in the sdpw-process regime (compare Figure 5a and Figure 5b). However, sdpw with 20 m/min at 3 kW and 4 kW shows higher feature values than sdpw with 20 m/min at 5 kW and 6 kW (Figure 5a), even though lower metallurgical weld widths result in lower power. However, this increase in the feature value relates to an increase in the measured OCT weld width of the interaction region. The increase in the measured width is assumed to be connected to an increase in melt pool and keyhole size at lower laser powers in the sdpw at the border to hcw. In this border region, the power is not sufficient to build up a fully formed keyhole, and the resulting denudation rather expands the melt pool in its width instead of the depth due to higher recoil pressure.

As soon as welds are in the hcw-process regime, feature values increase with increasing laser power (Figure 5a, 60 m/min). This can be explained by an increased measured OCT weld width due to increased energy input by higher laser powers. The increased thermal energy at higher laser powers leads to an increase in the melt pool size and, in consequence, to higher feature values. In the case of welding with the lowest laser power (3 kW), also a feature value result of 0 is possible. This feature value of 0 results from the measurement position behind the TCP. The molten area shrinks with lower laser power and results in a measurement of the surface topography behind the interaction region.

In conclusion, the influence of the laser power may influence the resulting feature value but does not show significant changes that might impact the classification task. The OCT measurement position behind the TCP allows feature values around 0 for hcw and shows the impact of OCT parameters on the feature value. In consequence, we further elaborate on the influence of OCT measurement parameters on the measurement result.

### 3.4. Limitations of Measurement Approach

Three different OCT measurement variables may affect the feature value: the measurement position, the frame rate, and the exposure time of the line sensor.

**Figure 6:**
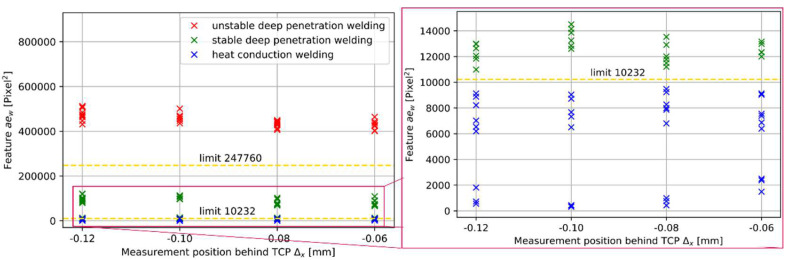
Feature values depending on relative OCT position behind TCP for different process regimes.

Four different measurement positions are considered behind the tool center point from 0.06 mm up to 0.12 mm for all welding conditions (Figure 6). No significant changes in the feature value can be found for udpw and hcw. Feature values for welds in the sdpw tend to show higher feature values for measurement positions further away from the TCP. This trend can be explained by an increased melt pool size behind the keyhole that tends to increase the measured width. The overall impact of the measurement position does not influence the identification of the weld category and hence plays a minor role in the identification of welding stability.

**Figure 7:**
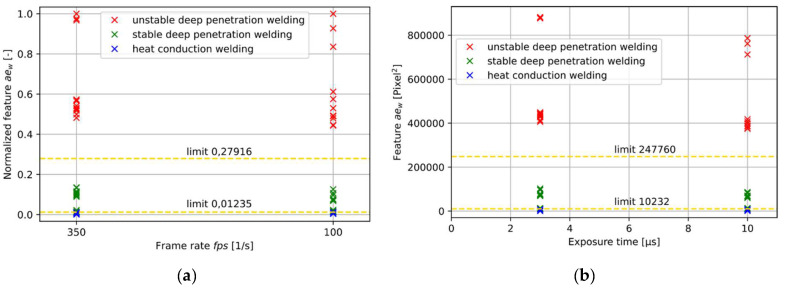
(**a**) Normalized feature value over different OCT frame rates for welds in different process regimes; (**b**) Feature value over different OCT exposure times for weld results in different process regimes. Categorization limits for different weld regimes can be seen as yellow dotted line.

A similar impact on the measurement result shows the variation of the exposure time from 3 µs to 10 µs (Figure 7a). A smaller exposure time leads to an increase in feature values. This can be explained by a slight increase in the number of frames for lower exposure times. A higher variation of the available number of frames by adjusting the frame rate. Results for frame rates at 87 Hz and 350 Hz can be seen in Figure 7b. The change in frame rate would lead to a significant change in feature values due to the change in the number of available frames. Normalization of the feature values with regard to the maximum feature value for each frame rate shows that the classification goal for hcw, sdpw, and udpw can be achieved for different frame rates (Figure 7a). A normalized feature value would allow for achieving more universal applicability of the feature value for the classification task.

## 4. Conclusions

In this paper, experimental work was performed with laser processing and OCT measurement parameters for the identification of different weld conditions based on OCT-measured width variations in the interaction region of laser welding of copper. The topography was measured inline to identify the width of the interaction region. The FRESH algorithm was used to select features for weld classification depending on the weld regime (stable/unstable deep penetration welding and heat conduction welding). The identified feature was discussed for the applicability of weld process categorization, changes in laser processing parameters, and possible limitations by the measurement method. It was shown that the classification of weld conditions is possible with the selected feature “absolute energy”. The measurement position along the weld seam and variations in laser power and welding speed did not result in significant changes with regard to the classification task. Changes in the frame rate show a significant impact on the feature value but can be avoided by normalization of the measurement results. Consequently, this enabled a proof-of-concept for inline monitoring of the weld stability based on surface topographical width measurements in the interaction region with OCT. We expect these findings to be beneficial in the future quality monitoring of laser welding. Future work will focus on an analysis of the impact of weld instabilities in the case of further weld imperfections and materials (e.g., cracks in the welding of high-strength aluminum alloys [23]). 

## Figures and Tables

**Table 1:** OCT parameters with parameter values.

OCT Parameters	Parameter Value
Measurement position behind TCP ∆_x_ (mm)	0.06, 0.08, 0.10, 0.12
Frame rate (Hz)	87, 350
Exposure time (µs)	3, 10

## Data Availability

Not applicable.
